# Single‐Neuron Responses to Odor‐Related Words in the Human Amygdala

**DOI:** 10.1111/ejn.70297

**Published:** 2025-11-10

**Authors:** Marlene Derner, Leila Chaieb, Roy Cox, Valeri Borger, Rainer Surges, Florian Mormann, Juergen Fell

**Affiliations:** ^1^ Department of Epileptology University Hospital Bonn Bonn Germany; ^2^ Netherlands Institute for Neuroscience Amsterdam Netherlands; ^3^ Department of Neurosurgery University Hospital Bonn Bonn Germany

**Keywords:** continuous recognition paradigm, medial temporal lobe, odor‐associated neurons, olfaction, single‐unit recordings

## Abstract

Human imaging studies suggest that visually presented words are processed by distributed networks beyond classical language areas, reflecting the properties related to their meanings. Based on human single‐neuron recordings, we investigated whether and how the odor aspect of words is processed in mediotemporal lobe regions involved in olfactory perception. We analyzed ensemble activity in response to odor‐related versus control words in the piriform cortex, amygdala, hippocampus, entorhinal cortex, and parahippocampal cortex and identified stimulus‐responsive and odor‐associated neurons. We detected converging evidence for odor‐associated responses to words in the amygdala, indicated by increased ensemble activity, and a significant proportion of odor‐associated neurons. These findings support and extend the notion that the amygdala integrates information across sensory modalities, allowing for the evaluation of its emotional and social significance.

## Graphical abstract

Based on human single‐neuron recordings, we investigated whether and how the odor aspect of words is processed in mediotemporal lobe regions involved in olfactory perception. We detected converging evidence for odor‐associated responses to words in the amygdala, indicated by increased ensemble activity, and a significant proportion of odor‐associated neurons. These findings support and extend the notion that different sensory inputs converge in the amygdala, allowing it to integrate information across sensory modalities.

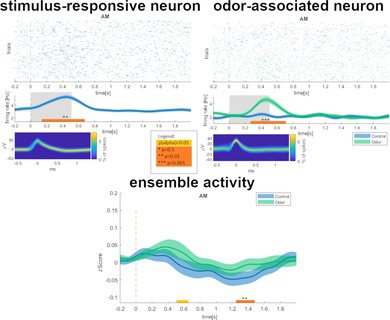

**Abbreviations**
fMRI: functional magnetic resonance imaging,
LSD: least significant differences

## 1 Introduction

The functional organization of odor perception is unique compared with other human senses (Gottfried 2010; Wilson and Sullivan 2011). Receptor neurons in the olfactory epithelium synapse to second‐order olfactory neurons in the olfactory bulb. The activity of these neurons directly projects to mediotemporal regions without first relaying through the thalamus. Human functional magnetic resonance imaging (fMRI) studies (e.g., Poellinger et al. 2001; Kjelvik et al. 2012; for a recent meta‐analysis, see Torske et al. 2022) and rodent single‐unit studies (Cain and Bindra 1972; Xu and Wilson 2012) have shown that the piriform cortex, amygdala, entorhinal cortex, and hippocampus are involved in odor processing. Recent findings based on human single‐unit recordings indicate that piriform neurons primarily encode chemical odor identity, while activity in the amygdala and hippocampus reflects subjective odor qualities (Kehl et al. 2024). Building on this approach, we investigated whether and how odor‐related words are represented by neurons in the human mediotemporal lobe. Previous fMRI studies suggest that visually presented words are processed by distributed networks beyond classical language regions that reveal the properties related to their meanings. For instance, action words have been shown to activate the premotor and motor cortices (Hauk, Johnsrude, et al. 2004), sound‐related words to engage the auditory cortex (Kiefer et al. 2008), and gustatory words to trigger responses in gustatory areas (Barrós‐Loscertales et al. 2012). More specifically, an fMRI study reported that odor‐related words elicit activations in the piriform cortex and amygdala (González et al. 2006). These findings are often conceptualized within the framework of embodied semantics, which addresses the question of whether, and to what degree, motor, and perceptual systems are involved in semantic representations and processes (Hauk and Tschentscher 2013). So far, results have been mixed, and views on this question remain controversial (e.g., Shebani and Pulvermüller 2013; Montero‐Melis et al. 2022; Mahon 2015).

In this study, we aimed to determine whether human single‐unit activity in the piriform cortex, amygdala, hippocampus, entorhinal cortex, and parahippocampal cortex reflects the multimodal processing of odor‐related words compared with control words. Specifically, the odor‐related words refer to objects that are sources of odors, but for brevity, we refer to them as odor‐related. To examine the evidence for odor‐related responses, we analyzed ensemble activity and identified stimulus‐responsive and odor‐associated neurons within these mediotemporal regions.

## 2 Materials and Methods

### 2.1 Participants

Recordings from five epilepsy patients (two females, three males; mean age: 47.4 ± 15.3 years) undergoing presurgical evaluation were analyzed. Mediotemporal depth electrodes and microwires had been implanted for chronic seizure monitoring and evaluation for epilepsy surgery. All patients gave informed written consent. The study conformed to the guidelines of the Medical Institutional Review Board at the University of Bonn (ethics vote numbers 095/10 and 248/11).

### 2.2 Selection of Odor‐Related and Control Words

**FIGURE 1:**
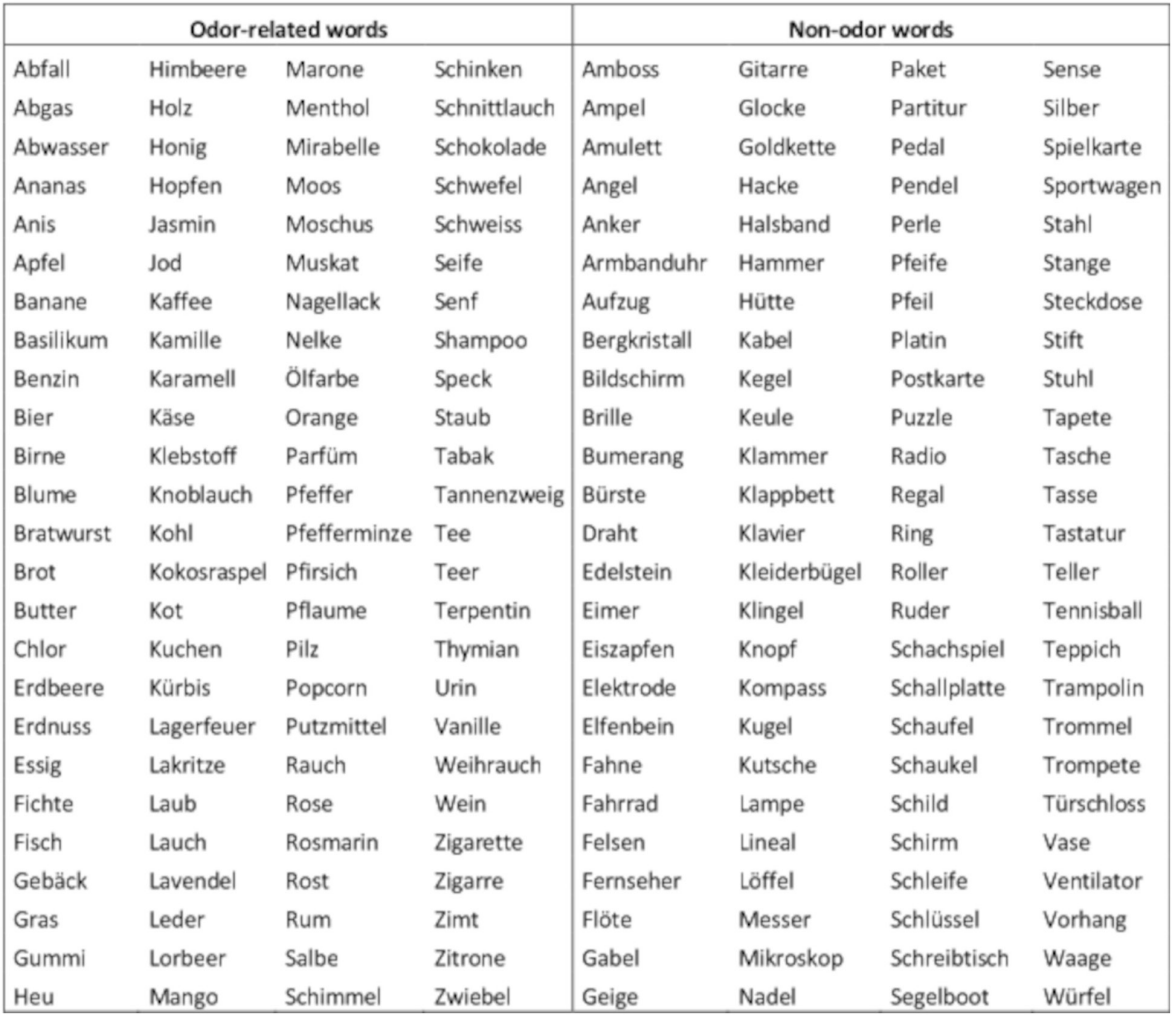
Word list. List of 200 selected German object words, including 100 odor‐related and 100 non‐odor‐related (control) words.

Two hundred German object words were selected, with 100 identified as odor‐related and 100 as non‐odor‐related (control) words. Examples of odor‐related words include “Abfall” (waste), “Ananas” (pineapple), “Benzin” (petrol), and “Blume” (flower). Examples of control words are “Ampel” (traffic light), “Angel” (fishing rod), “Brille” (glasses), and “Bürste” (brush) (for the full word list, see Figure 1). The odor‐related and control words were matched in terms of the number of syllables (mean ± SD: 2.1 ± 0.9 vs. 2.2 ± 0.6), word frequency (4.9 ± 7.1 vs. 6.1 ± 6.4), concreteness (6.7 ± 0.8 vs. 6.8 ± 0.8), arousal (3.7 ± 0.8 vs. 3.6 ± 0.9), imageability (6.5 ± 0.9 vs. 6.6 ± 0.8), and valence (5.4 ± 1.1 vs. 5.3 ± 0.7), with no significant differences found (unpaired *t*‐tests; all *p* > 0.2). Word frequency ratings (per million words) were extracted from the Digital Dictionary of the German Language (Digitales Wörterbuch der deutschen Sprache, DWDS 2020). Ratings for concreteness, arousal, imageability, and valence were obtained from a collection of 350,000 German words, assessed using a supervised learning algorithm (Köper and Schulte im Walde 2016). After alphabetically ordering the 200 object words, 12 healthy participants (eight females, four males; mean age: 33.1 ± 14.0) rated how strongly each word was associated with an odor on a discrete scale from 1 (no smell) to 10 (very strong smell). The odor‐related and control words differed highly significantly in their odor‐relatedness (5.6 ± 1.8 vs. 1.5 ± 0.5; unpaired t‐test: *p* < 0.0001).

### 2.3 Experimental Paradigm

A standard laptop running the Psychophysics Toolbox (Brainard 1997) under MATLAB (MathWorks Inc.) was used for stimulus presentation. Participants were asked to perform a continuous word recognition task (Fell et al. 2008). Each experimental run consisted of 300 words, with 100 shown once and 100 shown twice. The presented words were chosen from a pool of 200 words, half of which were identified as odor‐related and the other half as control words (see above: “Selection of odor‐related and control words”). The sequence of word stimuli was randomized within each run. Each word was displayed for 0.5 s, followed by a random post‐stimulus interval ranging from 1.7 to 2.3 s. After each word presentation, participants were required to indicate via a button press whether the word was shown for the first time (“new”) or had been presented before (“old”). If no response was registered by the end of the poststimulus interval, a “slow response” screen appeared and remained until the participant responded with either “old” or “new.”

### 2.4 Data Recording

Recordings were obtained from a bundle of nine microwires (eight high‐impedance recording electrodes, one low‐impedance reference, AdTech, Racine, WI) protruding from the end of each depth electrode targeting the hippocampus, entorhinal cortex, amygdala, parahippocampal cortex, and piriform cortex. Within the hippocampus, sections corresponding to the anterior (head) and posterior (body) hippocampus were targeted. Depth electrode placement was controlled by intraoperative computed tomography scans, co‐registering the head‐fixed stereotactic frame to preoperative MRI scans used for implantation planning. The differential signal from the microwires was amplified using a Neuralynx ATLAS system (Bozeman, MT), filtered between 0.1 and 9000 Hz, and sampled at 32.678 kHz. These recordings were stored digitally for further analysis. The number of recording microwires located within the mediotemporal lobe per patient was 96. Recording microwires were referenced against one of the reference microwires. Units from the left and right hemispheres were coded identically, with no distinction made between sides. Signals were band‐pass filtered between 300 and 3000 Hz. Spike detection and sorting were performed semiautomatically using the Combinato software (Niediek et al. 2016). As Combinato tends to overcluster the recorded unit data in automated mode, we manually merged clusters on the basis of their waveforms, cross‐correlograms, and other firing characteristics. Single‐unit recording quality and spike sorting were validated based on inter‐spike interval violations, spike amplitudes, and spike peak signal‐to‐noise ratio, as well as cluster isolation distance (Kehl et al. 2024). For statistical analysis, neurons were pooled across participants, which is standard practice in the field of human extracellular in vivo recordings.

### 2.5 Computation of Average Firing Rates

Data analysis was performed in MATLAB using the FieldTrip toolbox (Oostenveld et al. 2011). For the analysis of firing rates, trials corresponding to the first presentation of each word were selected. Spike counts were obtained for four non‐overlapping 500 ms bins, covering the time period from 0 to 2000 ms after stimulus onset, as well as for a baseline interval from −200 to 0 ms. Mean firing rates across trials were calculated for each time window, separately for odor‐word and control‐word trials, as well as across all trials. Normalized responses were then computed as *z*‐scored firing rates, based on the mean and standard deviation of firing rates during the baseline interval across all trials. Only units with a minimum mean firing rate of 2 Hz in at least one of the intervals were considered for further analysis. The mean firing rate (Hz) was calculated by counting the number of spikes within an interval across all first‐presentation trials, dividing this number by the number of first‐presentation trials (i.e., 200), and then multiplying this number by 2 (i.e., 1/0.5 s).

### 2.6 Statistical Evaluation of Ensemble Activity

Using IBM SPSS, mixed ANOVAs were conducted with the repeated‐measure factors ODOR (odor words, control words) and TIME (windows: [0; 500], [500; 1000], [1000; 1500], [1500; 2000]), as well as the independent factor REGION (hippocampus, entorhinal cortex, amygdala, parahippocampal cortex, piriform cortex) to explore the influences of odor‐relatedness, time, and brain region on single‐neuron responses. For post hoc comparisons, least significant difference (LSD) tests were calculated.

### 2.7 Identification of Stimulus‐Responsive Neurons

To identify stimulus‐responsive units, differences between *z*‐scored firing rates in the baseline interval versus the four post‐stimulus intervals were evaluated using paired *t*‐tests. Units with at least one *t*‐test showing a *p* value < 0.0125 (Bonferroni correction for four tests across four intervals) were considered significant. When the less conservative Benjamini–Hochberg correction was applied, only one additional stimulus‐responsive unit was identified (entorhinal cortex, increase post‐ vs. pre‐stimulus). To determine whether the number of units with statistically significant differences was higher than expected by chance, binomial tests with a probability of 0.05 (alpha level of 5%) were conducted, relating the number of stimulus‐responsive units to the total number of units in each region.

### 2.8 Identification of Odor‐Associated Neurons

To identify odor‐associated units, *z*‐scored firing rates from odor trials were compared with non‐odor trials within the same post‐stimulus time interval using two‐sided unpaired *t*‐tests. Again, units with at least one *t*‐test showing a *p*‐value < 0.0125 (Bonferroni correction for four intervals) were considered significant. The numbers of identified odor‐associated units remained the same when the less conservative Benjamini–Hochberg correction was applied. Additionally, binomial tests with a probability of 0.05 were performed to determine whether the number of odor‐related neurons was higher than expected by chance.

### 2.9 Data and Code Availability

In accordance with the ethics approval given by the ethics committee of the Medical Faculty of the University of Bonn and the guidelines of the German Research Foundation, pooled spiking data and program code will be made publicly available to researchers on a Github Online Repository (https://github.com/mderner/OdorContinuousRecognition). Further queries should be directly addressed to the corresponding author via email.

## 3 Results

Units in the targeted regions were identified based on semiautomatic spike detection and sorting, with manual supervision by an experienced expert (see “Materials and Methods”). In a subsequent step, only units with an average firing rate of at least 2 Hz across trials were retained for further analysis. This process resulted in 195 units from five brain regions: 57 in the amygdala (29%), 54 in the hippocampus (28%), 33 in the piriform cortex (17%), 29 in the parahippocampal cortex (15%), and 22 in the entorhinal cortex (11%). The following analyses are based on average *z*‐scored firing rates, using four consecutive post‐stimulus time windows, each with a duration of 500 ms.

### 3.1 Neurons Responsive to Object Words

**FIGURE 2:**
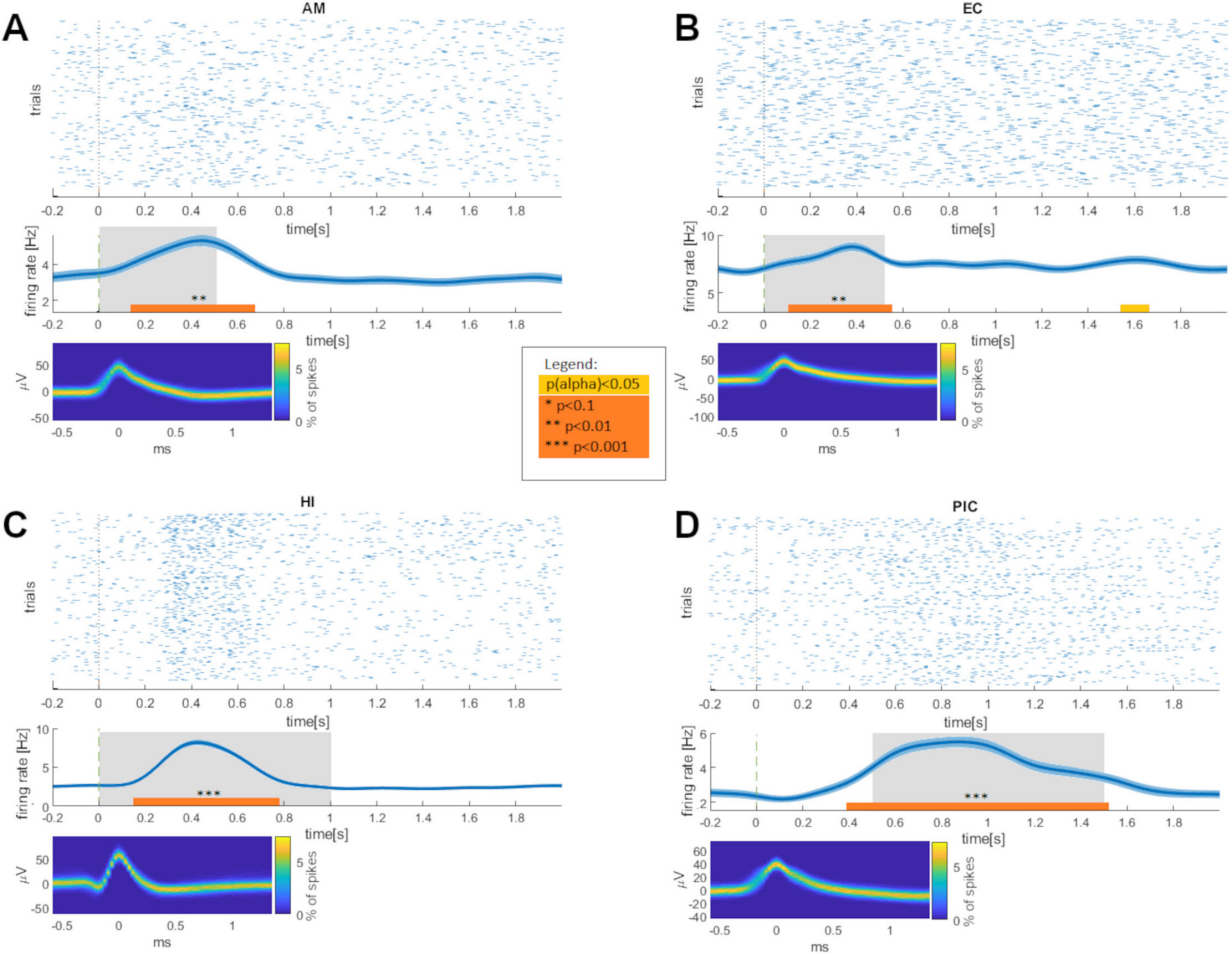
Examples of stimulus‐responsive neurons in different mediotemporal regions. (A–D) Four examples of stimulus‐responsive neurons are shown, with the respective regions indicated by abbreviations (AM, amygdala; EC, entorhinal cortex; HI, hippocampus; PIC, piriform cortex). Each subplot provides the following information: Top: Raster plot of spike times relative to stimulus onset (*t* = 0). Middle: Mean instantaneous firing rates (Hz) relative to stimulus onset. Shaded areas represent the standard error of the mean. Paired *t*‐tests were conducted for consecutive 500‐ms time intervals to compare firing rates during each of the four post‐stimulus intervals against the pre‐stimulus interval (−200 ms, 0 ms). Grey shaded areas indicate intervals with significant differences (Bonferroni‐corrected *p* < 0.0125). Additionally, bold horizontal lines represent time intervals identified by cluster permutation tests comparing post‐stimulus and pre‐stimulus firing rates (paired *t*‐tests; alpha threshold: *p* = 0.05; 10,000 permutations). Orange lines indicate significance at the cluster level, while beige lines indicate significance only at the alpha threshold. Bottom: Density plots of all spike waveforms. The plots show two‐dimensional histograms of spike voltages over time. The color code depicts the percentage of spikes (denominator: all spikes recorded for this unit) with the specified voltage at the given time point.

Application of two‐sided Bonferroni‐corrected *t*‐tests indicated that 33 of 195 units (17%) exhibited significantly different *z*‐scored firing rates in the post‐ versus pre‐stimulus intervals (most frequent interval (in 17 units): 500–1000 ms; see Table 1). Stimulus‐responsive neurons were most prevalent in the entorhinal cortex (27% of all entorhinal units, binomial test: *p* = 0.0006), amygdala (19%, *p* = 0.0001), and hippocampus (17%, *p* = 0.0013). Binomial tests showed only a trend for the piriform cortex (12%, *p* = 0.081) and indicated that the number of detected units in the parahippocampal cortex (10%; *p* = 0.175) was not above chance level. Results were identical for non‐*z*‐scored firing rates (see Figure 2A–D).

**TABLE 1:** Distribution of units responsive to object words in mediotemporal lobe regions. Percentage values refer to the total number of units in these regions.

Number of units	Amygdala	Hippocampus	Entorhinal cortex	Parahippocampal cortex	Piriform cortex
Total	57	54	22	29	33
Stimulus‐responsive	11 (19%)	9 (17%)	6 (27%)	3 (10%)	4 (12%)
Increase post versus pre	5	7	5	3	3
Decrease post versus pre	6	2	1	0	1
Binomial test: *p*‐value	0.0001	0.0013	0.0006	0.175	0.081

### 3.2 Odor‐Associated Neurons

**FIGURE 3:**
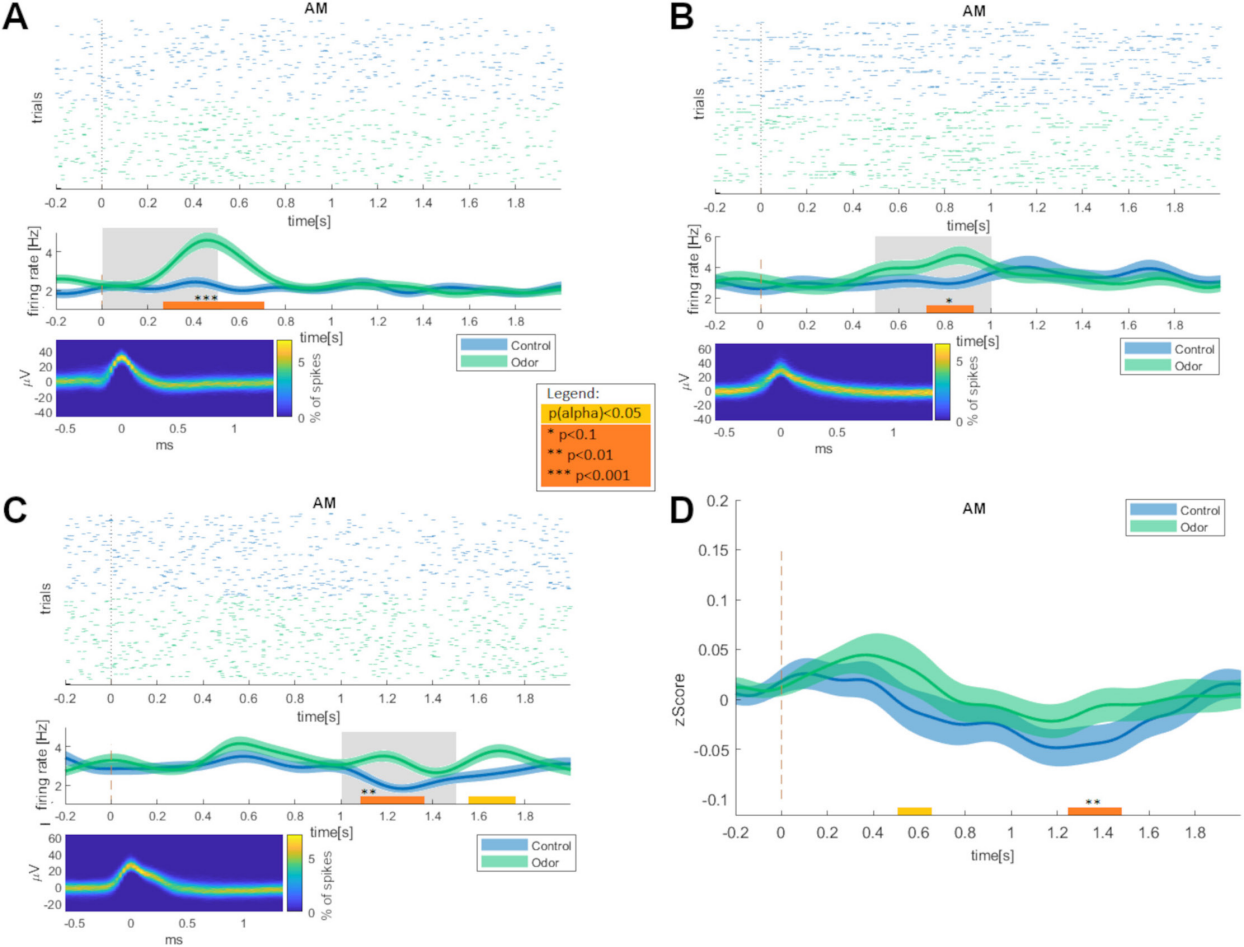
Examples of odor‐associated neurons and ensemble activity in the amygdala. (A–C) Three examples of odor‐associated units in the amygdala (abbreviation: AM). Each subplot provides the following information: Top: Raster plots of spike times relative to stimulus onset (*t* = 0) for odor‐related words (green) and control words (blue). Middle: Mean instantaneous firing rates (Hz) relative to stimulus onset for odor‐associated words (green) and control words (blue). Shaded areas represent the standard error of the mean. *T*‐tests were conducted for consecutive 500‐ms time intervals to compare firing rates for odor‐related versus control words. Grey shaded areas indicate intervals with significant differences (Bonferroni‐corrected for *N* = 4 time windows, *p* < 0.0125). Additionally, bold horizontal lines represent time intervals identified by cluster permutation tests comparing firing rates for odor‐related and control words (paired *t*‐tests; alpha threshold: *p* = 0.05; 10,000 permutations). Orange lines indicate significance or a trend toward significance at the cluster level, while beige lines indicate significance only at the alpha threshold. Bottom: Density plots of all spike waveforms. The plots show two‐dimensional histograms of spike voltages over time. The color code depicts the percentage of spikes (denominator: all spikes recorded for this unit) with the specified voltage at the given time point. (D) Ensemble activity averaged across amygdala neurons. The plot shows mean *z*‐scored firing rates across units relative to stimulus onset for odor‐related words (green) and control words (blue). Shaded areas represent the standard error of the mean. Bold horizontal lines represent time intervals identified by cluster permutation tests comparing firing rates for odor‐related and control words (paired *t*‐tests; alpha threshold: *p* = 0.05; 10,000 permutations). The orange line indicates significance at the cluster level (significant cluster for time interval [1239 ms, 1483 ms]: *p* = 0.0092), while the beige line indicates significance only at the alpha threshold.

Significantly different *z*‐scored firing rates between odor‐related versus control words (two‐sided Bonferroni‐corrected *t*‐tests) were observed for 11 of 195 units (6%; most frequent interval (in 7 units): 500–1000 ms; see Table 2). Odor‐associated neurons were significantly more prevalent than chance in the amygdala (12%, *p* = 0.023). The number of units detected in the other regions did not exceed chance level (entorhinal cortex: 5%, *p* = 0.68; hippocampus: 2%, *p* = 0.94; parahippocampal cortex: 3%, *p* = 0.77; piriform cortex: 3%, *p* = 0.50). Findings were identical for non‐*z*‐scored firing rates (see Figure 3A–C).

**TABLE 2:** Distribution of units with different responses to odor‐related versus control words in mediotemporal lobe regions.

Number of units	Amygdala	Hippocampus	Entorhinal cortex	Parahippocampal cortex	Piriform cortex
Total	57	54	22	29	33
Odor‐associated	7 (12%)	1 (2%)	1 (5%)	1 (3%)	1 (3%)
Increase odor versus cntr.	6	0	1	0	0
Decrease odor versus cntr.	1	1	0	1	1
Binomial test: *p*‐value	0.023	0.94	0.68	0.77	0.50

### 3.3 Ensemble Activity

To investigate whether ensemble responses to odor‐related words differ from control words, we conducted an ANOVA on *z*‐scored firing rates, with ODOR and TIME as repeated‐measures factors, and REGION as an independent factor. This ANOVA revealed a significant interaction between ODOR and REGION (*F*_4,190_ = 3.043, *p* = 0.018, partial eta‐squared η^2^ = 0.060). Post hoc LSD tests indicated higher *z*‐scored firing rates for odor‐related words compared with control words in the amygdala (mean ± SEM: 0.007 ± 0.013 vs. −0.012 ± 0.013, *p* = 0.008, Cohen’s *d* = 0.36; see Figure 3D), as well as lower *z*‐scores for odor‐related words versus control words in the piriform cortex (−0.027 ± 0.017 vs. −0.008 ± 0.017, *p* = 0.038, Cohen’s *d* = 0.37). In the other regions, *z*‐scored firing rates did not differ significantly (hippocampus: 0.024 ± 0.013 vs. 0.019 ± 0.013; parahippocampal cortex: 0.037 ± 0.018 vs. 0.042 ± 0.018; entorhinal cortex: 0.028 ± 0.021 vs. 0.036 ± 0.020). Moreover, there were no other significant ANOVA main effects or interactions.

To assess the robustness of these results, we also conducted an ANOVA of non‐*z*‐scored firing rates, which yielded similar findings. There was a significant interaction between ODOR and REGION (*F*_4,190_ = 2.571, *p* = 0.039, partial eta‐squared η^2^ = 0.051). Additionally, post hoc LSD tests revealed higher firing rates for odor‐related versus control words in the amygdala (3.50 ± 0.41 Hz vs. 3.41 ± 0.42 Hz, *p* = 0.018, Cohen’s *d* = 0.32), and a trend toward lower firing rates in the piriform cortex (2.61 ± 0.54 vs. 2.70 ± 0.55, *p* = 0.062, Cohen’s *d* = 0.33).

To obtain more detailed information on the time course of the odor effect in the amygdala (see Figure 3D), we conducted an additional ANOVA on *z*‐scored firing rates using a higher temporal resolution with 100 ms windows. As mathematically expected, this ANOVA again yielded the significant interaction between ODOR and REGION (*F*_4,190_ = 3.043, *p* = 0.018, partial eta‐squared η^2^ = 0.060). Furthermore, there was a trend toward a three‐way interaction between ODOR, REGION, and TIME (*F*_76,3610_ = 1.232, *p* = 0.085, partial eta‐squared η^2^ = 0.025). Exploratory post hoc LSD‐tests showed significantly increased *z*‐scores in the amygdala for odor‐related compared with control words in the 500–600‐ms time window (0.037 ± 0.032 vs. −0.037 ± 0.028, *p* = 0.010, Cohen’s *d* = 0.35), as well as a trend toward an increase in the 1300–1400‐ms time window (0.015 ± 0.025 vs. ‐0.029 ± 0.023, *p* = 0.092, Cohen’s *d* = 0.22).

One may wonder whether the frequently examined contrast between living and nonliving word categories may have influenced our results (Binder et al. 2009), given that the odor‐related words are more often associated with the living category than the control words. To assess this potential bias, we classified the presented words into living versus nonliving categories and conducted an ANOVA on *z*‐scored firing rates, with LIVING and TIME (500 ms windows) as repeated‐measures factors, and REGION as an independent factor. This ANOVA revealed a significant interaction between LIVING and REGION (*F*_4,190_ = 6.090, *p* < 0.001, partial eta‐squared η^2^ = 0.114). However, post hoc LSD tests showed a pattern differing from the ODOR * REGION interaction. Specifically, *z*‐scored firing rates were lower for the living versus nonliving category in the hippocampus (mean ± SEM: 0.002 ± 0.014 vs. 0.037 ± 0.014, *p* = 0.001, Cohen’s *d* = 0.42), whereas the odor versus non‐odor contrast showed no effect in the hippocampus. In the piriform cortex, *z*‐scores were higher for the living versus nonliving category (0.003 ± 0.018 vs. −0.045 ± 0.018, *p* < 0.001, Cohen’s *d* = 0.61), whereas they were lower for the odor versus non‐odor contrast. In the other regions, *z*‐scored firing rates did not differ significantly (amygdala: *p* = 0.590; entorhinal cortex: *p* = 0.919; parahippocampal cortex: *p* = 0.209), and no other significant main effects or interactions were observed in the ANOVA. These findings indicate that our results concerning the odor aspect of words cannot be explained by a living versus nonliving bias.

We further examined whether the food versus nonfood contrast may have contributed to our findings by conducting an additional ANOVA on *z*‐scored firing rates, with FOOD and TIME as repeated‐measures factors and REGION as an independent factor. This analysis again revealed a significant interaction between FOOD and REGION (*F*_4,190_ = 3.540, *p* = 0.008, partial eta‐squared η^2^ = 0.069). Post hoc LSD tests showed higher *z*‐scores for food versus nonfood in the amygdala (mean ± SEM: 0.014 ± 0.014 vs. −0.008 ± 0.012, *p* = 0.008, Cohen’s *d* = 0.36), as well as a trend toward lower *z*‐scores in the hippocampus (mean ± SEM: 0.010 ± 0.015 vs. 0.026 ± 0.013, *p* = 0.069, Cohen’s *d* = 0.26). There was no effect in the piriform cortex (−0.014 ± 0.019 vs. −0.019 ± 0.016; *p* = 0.628) or any other region (entorhinal cortex: *p* = 0.166; parahippocampal cortex: *p* = 0.225). Thus, both the food versus nonfood and odor versus non‐odor contrasts are associated with increased *z*‐scored firing rates in the amygdala. However, the overall response patterns differ: The hippocampus showed a trend toward decreased firing for food versus nonfood words, whereas the piriform cortex showed a significant decrease for odor versus non‐odor words. Importantly, an additional two‐way ANOVA on *z*‐scored firing rates for odor words in the amygdala, with FOOD (food vs. nonfood) and TIME as repeated measures factors, did not yield a significant main effect of FOOD (*p* = 0.111; *F*_1,56_ = 2.628). These findings suggest that the observed effects related to the odor aspect of words cannot readily be explained by the food versus nonfood contrast.

## 4 Discussion

Based on the analysis of single‐unit activity in mediotemporal brain regions, we found converging evidence for odor‐associated responses to words in the human amygdala. Evaluation of ensemble activity revealed increased firing rates following the visual presentation of odor‐related compared with non‐odor‐related object words in the amygdala. Additionally, examination of individual neurons showed that a statistically significant proportion (12%) of neurons in the amygdala exhibited differential responses to odor‐related versus control words. While a statistically significant number of neurons in the amygdala, hippocampus, and entorhinal cortex responded to object words in general, above‐chance responses to the odor aspect of the words were observed only in the amygdala.

Our findings align well with the idea that different sensory inputs converge in the amygdala, allowing it to integrate information across sensory modalities (Uwano et al. 1995; Kuraoka and Nakamura 2006; Quian Quiroga et al. 2009; Morrow et al. 2019; Kehl et al. 2024). This integration may support the evaluation of the emotional and social significance of incoming signals (Rutishauser et al. 2015), particularly through the combination of visual and olfactory information (Novak et al. 2015). Supporting this view, a substantial proportion of amygdala neurons in rats have been shown to respond to multimodal sensory stimuli, including visual and olfactory ones (Uwano et al. 1995). Interestingly, fMRI responses in the amygdala to food stimuli have been found to increase when the odor stimulus was removed during bimodal stimulation with an odor and an image (Schicker et al. 2022).

A recent single‐neuron study described odor representations in the human mediotemporal lobe (Kehl et al. 2024). This study also investigated the processing of odor‐related images. In line with our current data, a significant proportion of neurons in the amygdala responded to the odor aspect of an image (Kehl et al. 2024), as did a highly significant proportion of neurons in the piriform cortex. In the piriform cortex, decoding of the odor aspect generalized when training on odors and testing on images, though not vice versa. However, in the amygdala, decoding generalized in both directions. Here, we found no evidence for odor‐associated neurons in the piriform cortex responding to odor‐related words. Speculatively, this divergence may stem from the fact that the processing of pictorial odor information is evolutionarily older than that of semantic odor information. Another speculative idea is that the olfactory system may have adapted to downregulate piriform activity in response to odor‐related object words (Franks and Isaacson 2005), as such words (e.g., the word “rose”) rarely signal the presence of an actual odor and do not closely resemble perceptual experiences in which olfactory input is expected. In contrast, images of odor‐related objects (e.g., the vivid image of a rose) may more strongly resemble such perceptual experiences, where visual and olfactory information often co‐occur. This closer resemblance might make it more difficult for the system to apply such downregulation for images of odor‐related objects. Nevertheless, neurons in the piriform cortex and amygdala have also been shown to respond to specific, repeatedly presented odor‐related images and words (Kehl et al. 2024).

Another possibility is that the olfactory system has adapted to suppress piriform activity in response to odor‐related words, which, unlike real odors, rarely signal the presence of an actual olfactory stimulus. In contrast, visual images of odor‐associated objects (e.g., a rose or a cup of coffee) may more strongly resemble real‐world perceptual experiences in which odors are often present. This greater ecological validity might make it more difficult for the brain to downregulate piriform activity in response to such stimuli.

An fMRI study by González et al. (2006) investigated the processing of odor‐related versus control words, reporting significant odor‐related activation in the amygdala and piriform cortex using small volume correction. However, odor‐related and control words were matched only for word length and frequency. In our study, we further matched words for concreteness, arousal, imageability, and valence. We observed increased ensemble activity in the amygdala but decreased activity in the piriform cortex in response to odor‐related versus control words. Notably, the relationship between fMRI responses and neuronal ensemble activity is complex. For example, fMRI activation may reflect the activity of excitatory or inhibitory neurons (Poplawsky et al. 2021; Moon et al. 2021), and the latter could hypothetically lead to a decrease in ensemble responses.

It has been argued that temporal information is crucial for understanding the neural representation and processing of semantics (Hauk 2016). Regarding the timing of increased odor‐related ensemble responses to words in the amygdala, our findings tentatively suggest an initial response occurring between 500 and 600 ms after stimulus onset. This time range corresponds to the latter part of the event‐related N400 component, which is widely regarded as a marker of semantic processing (Kutas and Federmeier 2011). Although earlier differences—even before 200 ms—in responses to different semantic word categories have been reported in event‐related potentials (Hauk, Coutout, et al. 2012; Amsel et al. 2013), such early odor‐related differences are not evident in our data. Within the context of the embodied semantics framework, our findings are consistent with the idea of embodiment, insofar as the increased odor‐related ensemble responses in the amygdala, a multimodal region, suggest that semantic information is not represented and processed solely at a modality‐independent level (Hauk and Tschentscher 2013; Mahon 2015).

In conclusion, our data provide direct evidence that neurons in the human amygdala respond to the odor aspect of visually presented words.

## Author Contribution

**Marlene Derner:** formal analysis, software, writing – original draft, writing – review and editing. **Leila Chaieb:** investigation, writing – original draft, writing – review and editing. **Roy Cox:** conceptualization, software, writing – original draft. **Valeri Borger:** resources, writing – original draft. **Rainer Surges:** resources, writing – original draft. **Florian Mormann:** conceptualization, methodology, writing – original draft. **Juergen Fell:** conceptualization, methodology, formal analysis, writing – original draft, writing – review and editing, supervision.

## Conflicts of Interest

The authors declare no conflicts of interest.

## Data Availability

The data that support the findings of this study are available from the corresponding author upon reasonable request.
